# Preliminary Analysis of Modified Low-Density Lipoproteins in the Serum of Healthy and Obese Dogs and Cats

**DOI:** 10.3389/fvets.2015.00034

**Published:** 2015-09-17

**Authors:** Nobuko Mori, Yuki Okada, Naoto Tsuchida, Yutaka Hatano, Makoto Habara, Shingo Ishikawa, Ichiro Yamamoto, Toshiro Arai

**Affiliations:** ^1^Department of Basic Veterinary Medicine, School of Veterinary Medicine, Nippon Veterinary and Life Science University, Tokyo, Japan

**Keywords:** oxidized LDL, dog, cat, ELISA, electrophoresis

## Abstract

Oxidized low-density lipoprotein (LDL) is thought to play an important role in the inflammatory response associated with human obesity. The purpose of this preliminary study was to determine oxidized LDL concentrations in healthy dogs and cats, and to evaluate whether obesity affects oxidized LDL concentration, using 39 cats and 19 dogs that had visited two different veterinary clinics in Japan. We hypothesized that oxidized LDL concentrations measured against body condition score (BCS) may have a potential value in evaluating the qualities of accumulated or circulating lipids in obese dogs and cats that do not show signs of metabolic diseases. The mean oxidized LDL value in BCS3 dogs (2.4 ± 0.9 μg/dl) was very similar to that of BCS5 dogs (2.2 ± 0.3 μg/dl). The mean oxidized LDL value of BCS4 dogs was 7.2 ± 10.3 μg/dl and the highest among three groups. BCS4 dogs included two dogs whose oxidized LDL values were higher than the mean oxidized LDL value of healthy humans (11.2 ± 0.3 μg/dl). On the other hand, the mean oxidized LDL value of BCS3 cats was 2.5 ± 0.9 μg/dl, and those of BCS4 and 5 cats were higher than that of BCS3, but there was no significant difference. The BCS4 cat group included one cat with a higher oxidized LDL value, and the BCS5 group also included two cats with oxidized LDL values higher than the mean oxidized LDL value of healthy humans. Interestingly, the oxidized LDL values in two obese dogs and three obese cats were indeed higher than the mean oxidized LDL value of humans with coronary artery disease (20.1 ± 1.1 μg/dl). In conclusion, this preliminary study showed reference ranges of oxidized dogs and cats against BCS. Obesity alone does not appear to have any direct effect on serum oxidized LDL values in healthy dogs and cats.

## Introduction

Hyperlipidemia can be characterized by quantitative increases in circulating lipoproteins or by higher lipid concentrations of various lipoproteins classes. Obesity in dogs and cats is commonly associated with hyperlipidemia. Generally, the treatment of hyperlipidemia in dogs and cats is to reduce the intake of cholesterol and/or triglycerides (TG). Recently, the modification of lipids has been identified as an important factor in human hyperlipidemia; therefore, it has been considered more effective to inhibit the oxidization of low-density lipoprotein (LDL) rather than to reduce the amount of cholesterol in humans (1). For instance, oxidized LDL is thought to play an important role in the inflammatory response. Oxidized LDL alters the biological properties of growth factors, cytokines, monocytes, and T lymphocytes by modulating their production (2–8). Acute coronary syndrome is triggered by the activation of immune-mediated inflammatory processes with atherothrombosis (9–11). Some reports indicated that highly oxidized LDL has been detected in the plasma of coronary heart disease (CHD) patients (12–14). Studies in laboratory animals and humans have shown that oxidized LDL is a better predictor of atherosclerosis and cardiovascular disease. In animal studies, administration of antioxidant drugs, like probucol, inhibits LDL oxidation and arterial plaque formation, even when there is no change in blood cholesterol levels (15–18). In fact, administration of the antioxidant butylated hydroxytoluene (BHT) significantly reduces the degree of atherosclerosis in the aorta of rabbits, even though it raises LDL cholesterol levels (19).

By immunoassays, using murine monoclonal antibodies prepared against malondialdehyde-modified LDL (MDA-LDL) found in human plasma, higher concentrations of MDA-LDL were found in the patients with CHD (13, 20). Elevated concentrations of oxidized LDL are predictive of future CHD events in apparently healthy men (21). Because CHD is the leading cause of human death, there are many studies of oxidized LDL causing CHD and/or arteriosclerosis. It was shown that an increase in oxidized LDL level is the most important factor associated with CHD (22).

It is commonly considered that dogs and cats rarely develop atherosclerosis in the absence of metabolic disease (23). This is due to the fact that high-density lipoprotein (HDL) levels in dogs and cats are higher than LDL, VLDL, and chylomicron levels. They are considered to be HDL-dominant animals unlike humans (24). Therefore, many veterinarians do not test for oxidized LDL levels in dogs and cats. There are no oxidized LDL data of dogs and cats with/without disease. There is no data available to evaluate whether high concentrations of LDL or oxidized LDL are risk factors for CHD.

However, this does not mean that dogs and cats do not develop atherosclerosis. It has been reported that dogs develop severe atherosclerosis when a plasma cholesterol concentration of over 750 mg/dl persists for more than 6 months (25). Some veterinarians feel that as obesity in animals has increased in developed countries, so will atherosclerosis, as seen in humans.

A retrospective report by Hess (26) showed that dogs with atherosclerosis were over 53 times more likely to have concurrent diabetes mellitus than dogs without atherosclerosis. By the same token, dogs with atherosclerosis were over 51 times more likely to have concurrent hypothyroidism than dogs without atherosclerosis (26). Other studies have shown that glomerulopathy is more prevalent in dogs with atherosclerosis compared to dogs without atherosclerosis (27). If we could accurately measure and understand oxidized LDL concentrations in dogs and cats, it may become a preventive marker for metabolic syndrome, diabetes mellitus, hypothyroidism, and glomerulopathy. The purpose of this preliminary study was to investigate oxidized LDL concentrations and develop the normal ranges of oxidized LDL concentration in healthy dogs and cats, and to evaluate how obesity affects the concentrations of oxidized LDL.

Obesity is a clinical sign that develops as the first sign of metabolic disease. When dogs and cats gain weight, their lipoprotein fractions are modified as well. Previously, we showed a specific elevation in HDL_1_ fraction, which plays an important role in reverse cholesterol transport similar to LDL, in obese dogs, and increased LDL concentrations of all fractions in obese cats (24). Therefore, we hypothesized that oxidized LDL concentration measured against body condition score (BCS) may have a potential value in evaluating the qualities of accumulated or circulating lipids in obese dogs and cats that do not show clinical signs of metabolic diseases.

We selected the following two methods to measure and analyze oxidized LDL: one was to determine the quantity of oxidized LDL by ELISA, and the second method was to validate modified LDL (modified charged LDL) by lipoprotein electrophoresis customized for dogs and cats.

## Materials and Methods

### Animals

This study consisted of 19 dogs and 39 cats that had visited two different veterinary clinics (Ibaraki and Kanagawa) during a 2-month period from the middle of November 2014 to the middle of January 2015. The dogs utilized in the study included 8 females and 11 males, ages 1–16 years old, and the cat group consisted of 22 females and 17 males, ages 0–15 years old. The dog breeds represented were American cocker spaniel [female (F) *n* = 1, male (M) *n* = 2], Chihuahua (F-1, M-1), Labrador retriever (M-1), Maltese (M-1), Miniature dachshund (F-1, M-2), Shiba (F-1), Shih tzu (F-1, M-1), Toy poodle (F-2), Yorkshire Terrier (M-1), and Mixed breed (F-1, M-2). The cat group included American shorthair (M-1), British shorthair (M-1), Norwegian (F-2, M-1), Scottish Fold (M-1), and Mixed breed (F-20, M-13). The subjects were diagnosed to be clinically healthy, according to the physical examination, clinical signs, and biochemical data evaluated by the attending veterinarians. These subjects did not have hypothyroidism or diabetes mellitus. They received a BCS of 1–5 (28) based on palpation and inspection. The BCS applies a 5-point scale: (1, very thin; 2, underweight; 3, ideal; 4, overweight; 5, obese). The resultant groups of dogs and cats were classified as Normal (BCS3) and Obese (BCS4, 5).

This study was approved by the Nippon Veterinary and Life Science University Animal Research Committee.

### Blood sampling

Postprandial blood samples (at least 4 h after the last meal) were collected from the cephalic veins into serum separator plastic tubes (Venoject II, Terumo Corporation, Tokyo, Japan). They were centrifuged at approximately 300 rpm for 15 min. In accordance with an Ox LDL ELISA kit protocol, all samples were aliquotted for oxidized LDL measurement to avoid loss of bioactivity and contamination, and were stored at −20°C for no more than 2 months. They were not processed through repeated freeze–thaw cycles.

### Analysis of serum parameters

Serum lipid metabolites and enzyme activities were measured. Glucose (GLU), TG, total cholesterol (T-Cho), alanine aminotransferase (ALT), aspartate aminotransferase (AST), total protein, blood urea nitrogen (BUN), and creatinine (CRE) were determined using a biochemical auto analyzer (JCA-BM2250, JEOL Ltd., Tokyo, Japan) with the manufacturer’s reagents. Oxidized LDL was determined using a Dog Oxidized low-density lipoprotein ELISA (Kameyama Biomedical Company, WA, USA). This ELISA kit uses a mouse monoclonal antibody for all of malondialdehyde (MDA)-LDL, Nepsilon (carboxymethyl) lysine (CML)-LDL, and detects advanced glycosylation end product (AGE)-LDL; it cannot distinguish between them. We constructed a calibration curve by plotting the average optical density for each calibrator on the horizontal (*X*) axis against the concentration on the vertical (*Y*) axis, and drawing the best-fit curve to generate a four parameter logistic (4-PL) curve-fit.

### Electrophoresis of lipoprotein cholesterol

Serum lipoprotein cholesterol profiles were measured by the biphasic agarose gel electrophoresis method using a commercial kit, Quick gel LIPO gels (Helena Laboratories, Saitama, Japan). Agarose gel fraction bands were analyzed using customized Epalyzer 2 Electrophoresis Processing Analyzer (Helena Laboratories, Saitama, Japan). In brief, 30 μl of each sample was loaded onto the gel, and run with a 14-min migration time at 250 V and 20°C. After migration, the gels underwent a 15-min reaction time, followed by a 12-min decolorizing and a 30-s fixing time. Cholesterol lipoprotein fractions were assessed and analyzed using Edbank III analysis software (Helena Laboratories, Saitama, Japan).

The band of modified LDL on electrophoresis migrates toward the positive electrode when the charge of apo-lipoprotein in LDL is modified. We set normal animals (BCS3) as control and compared the band position of obese animals against each control group.

### Statistical analysis

Values are expressed as mean ± SD. Statistical significance was determined by Holm–Sidak One-way ANOVA. All tests were performed using Sigmaplot version.11.2 (Systat Software Inc., San Diego, CA, USA). The significance level was set at *p* < 0.05. The correlations were determined by Pearson’s product-moment correlation coefficients, *r*, and are displayed as *p* < 0.05. The Electrophoresis bands were assessed and analyzed using Edbank III analysis software (Helena Laboratories, Saitama, Japan).

## Results

**Figure 1:**
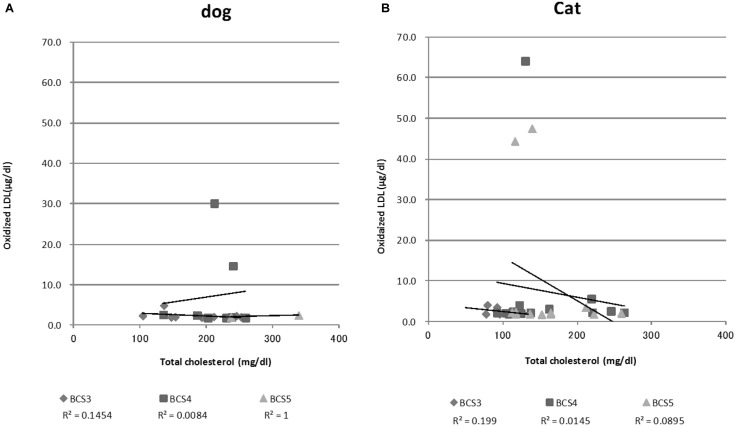
**Serum oxidized LDL and total cholesterol concentrations among all dogs (A) and cats (B)**. This graph shows a correlation between total cholesterol levels and oxidized LDL levels by Pearson’s product-moment correlation.

The parameters measured in normal (BCS3) and obese (BCS4 and 5) animals are shown in Table 1. The T-Cho of BCS5 dogs was significantly higher than that of BCS3 dogs, but other parameters did not show significant differences. On the other hand, cats showed significant differences in certain parameters. Age, BW, T-Cho, and total protein of BCS4 and 5 cats were higher than those of BCS3 cats. The ALT of BCS4 cats was significantly lower than that of BCS3 cats, whereas the CRE value of BCS4 cats was higher than that of BCS3 cats. Figure 1 shows the correlations of T-Cho and oxidized LDL with BCS groups. Dog oxidized LDLs that correlated with T-Cho were BCS3 (*r* = −0.4), BCS4 (*r* = 0.1), and BCS5 (*r* = 1.0). Cat oxidized LDLs that correlated with T-Cho were BCS3 (*r* = −0.4), BCS4 (*r* = −0.1), and BCS5 (*r* = −0.3).

**Table 1:** **Comparison of parameters in normal and obese animals**.

Dog	Normal	Obesity		High oxidized LDL dogs in obesity (*n* = 2)
	BCS3 dog (*n* = 9)	BCS4 dog (*n* = 8)	BCS5 dog (*n* = 2)	
Age (years)	6.5 ± 3.6	9.0 ± 6.4	12.5 ± 0.7	13.5 ± 0.7
BW (kg)	5.6 ± 2.8	13.1 ± 10.2	14.0 ± 7.0	18.9 ± 13.9
BCS (/5)	3.2 ± 0.3	4.0 ± 0.0^a^	5.0 ± 0.0^a^	4.0 ± 0.0
Glucose (mg/dl)	91.4 ± 43.2	77.7 ± 33.1	54.5 ± 6.4	62.5 ± 44.5
Triglyceride (mg/dl)	72.4 ± 64.1	127.1 ± 106.3	489.0 ± 367.7	195.5 ± 77.1
Total cholesterol (mg/dl)	183.0 ± 50.8	212.8 ± 39.1	288.0 ± 72.1^a^	226.0 ± 19.8
Oxidized LDL (μg/dl)	2.4 ± 0.9	7.2 ± 10.3	2.2 ± 0.3	22.4 ± 11.0
ALT (IU/l)	56.1 ± 31.0	63.0 ± 53.6	50.5 ± 44.5	112.5 ± 98.3
AST (IU/l)	35.2 ± 8.3	33.4 ± 14.8	23.5 ± 3.5	33.5 ± 12.0
Total protein (g/dl)	7.1 ± 0.7	7.4 ± 0.8	7.9 ± 0.9	7.6 ± 0.6
BUN (mg/dl)	17.2 ± 6.4	17.0 ± 4.4	17.5 ± 4.9	19.0 ± 9.9
Creatinine (mg/dl)	0.8 ± 0.2	1.0 ± 0.4	1.1 ± 0.6	1.2 ± 0.5

*Values are presented as means ± SD. BCS, body condition score*.

High oxidized LDL dogs in obesity is animals with mean value of oxidized LDL greater than that of humans (11.2 ± 0.3 μg/dl)

*^a^Indicates significance (Holm–Sidak One-way ANOVA, *p* < 0.05) when BCS4 or 5 High ox LDL dogs were compared pairwise against BCS3 dog*.

**Table:** 

Cat	Normal	Obesity		High oxidized LDL cats in obesity (*n* = 3)
	BCS3 cat (*n* = 10)	BCS4 cat (*n* = 15)	BCS5 cat (*n* = 14)	
Age (years)	1.0 ± 1.2	6.9 ± 5.1^a^	8.7 ± 4.7^a^	12.3 ± 3.8
BW (kg)	3.1 ± 0.7	4.7 ± 0.8^a^	6.7 ± 2.2^a^	6.0 ± 2.0
BCS (/5)	3.0 ± 0.0	4.0 ± 0.0^a^,^b^	5.0 ± 0.0^a^	4.7 ± 0.6
Glucorse (mg/dl)	168.4 ± 43.0	130.2 ± 57.9	122.5 ± 70.9	153.0 ± 35.4
Triglyceride (mg/dl)	37.5 ± 19.3	66.1 ± 41.8	152.6 ± 218.3	71.7 ± 11.6
Total cholesterol (mg/dl)	96.7 ± 24.9	158.5 ± 55.9^a^	168.1 ± 50.0^a^	128.3 ± 11.6
Oxidized LDL (μg/dl)	2.5 ± 0.9	7.5 ± 17.0	9.5 ± 17.1	52.0 ± 10.6
ALT (IU/l)	73.0 ± 38.9	36.7 ± 18.8^a^	47.8 ± 30.4	54.5 ± 7.8
AST (IU/l)	32.4 ± 17.4	24.4 ± 19.0	31.1 ± 20.4	23.5 ± 2.1
Total protein (g/dl)	7.3 ± 0.4	7.9 ± 0.6^a^	8.2 ± 0.9^a^	8.9 ± 0.1
BUN (mg/dl)	22.4 ± 4.3	25.0 ± 4.0	28.4 ± 18.1	20.5 ± 2.1
Creatinine (mg/dl)	1.2 ± 0.3	1.5 ± 0.3^a^	1.4 ± 0.3	1.6 ± 0.1

*Values are presented as means ± SD. BCS, body condition score*.

High oxidized LDL cats in obesity is animals with mean value of oxidized LDL greater than that of humans (11.2 ± 0.3 μg/dl)

*^a^Indicates significance (Holm–Sidak One-way ANOVA, *p* < 0.05) when BCS4 or 5 cats were compared pairwise against BCS3 cat*.

*^b^Indicates significance (Holm–Sidak One-way ANOVA, *p* < 0.05) when BCS4 cat were compared pairwise against BCS5 cat*.

The oxidized LDL value in BCS3 dogs (2.4 ± 0.9 μg/dl) was similar to BCS5 dogs (2.2 ± 0.3 μg/dl). The oxidized LDL value of BCS4 dogs (7.2 ± 10.3 μg/dl) tended to be higher than other BCS groups. The BCS4 group included two dogs whose oxidized LDL values were higher than that of healthy humans (11.2 ± 0.3 μg/dl) (29). The group consisted of one Mixed breed (14-year-old female) and one Miniature dachshund (13-year-old male).

On the other hand, the mean oxidized LDL value of BCS3 cats was 2.5 ± 0.9 μg/dl, BCS4 was 7.5 ± 17.0 μg/dl, and BCS5 was 9.5 ± 17.1 μg/dl; however, these increases were not significant. BCS4 and 5 cats also included three cats with oxidized LDL values that were higher than that of healthy humans. These cats were Mixed breed (8-year-old male, 14-year-old male, 15-year-old female). Figure 1 also shows the list of oxidized LDL dogs and cats that were extremely high.

**Figure 2:**
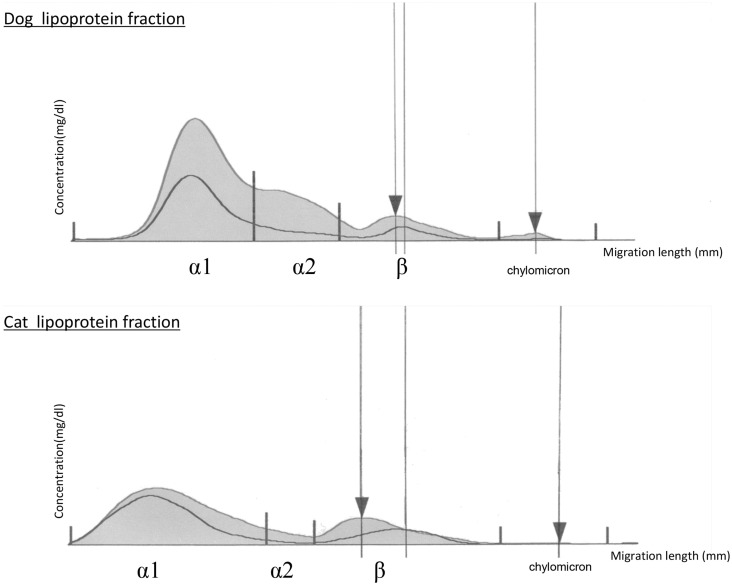
**Graphs of lipoprotein cholesterol separated by electrophoresis in obese dogs and cats**. The upper curve represents the obese animal lipoprotein cholesterol fraction, and the lower curve represents a normal animal lipoprotein cholesterol fraction. Left ▼ is the LDL peak of obese animals. Right ▼ represents the origin. The electrophoresis bands were assessed and analyzed using Edbank III analysis software (Helena Laboratories, Saitama, Japan) and shown graphically.

When we examined the lipoprotein fractions by electrophoresis, one obese dog and six obese cats had LDL peaks migrating toward the positive electrode, unlike that of the controls. Representative examples of degeneration fraction graphs with the LDL migration of dogs and cats were as seen in Figure 2. The results were not consistent with the quantitative measurement of oxidized LDL. In fact, seven animals that had LDL fractions migrating toward the positive side on electrophoresis did not have higher oxidized LDL values on ELISA.

## Discussion

Previous studies have shown that thyroid dysfunction is accompanied by changes in caloric intake and body weight, as well as changes in the lipid profile. Hypothyroid rats showed significantly decreased oxidized LDL levels, whereas hyperthyroid rats showed significantly increased oxidized LDL levels (30). Another report showed that oxidized LDL levels in 125 obese human patients were positively correlated with body mass index and T-Cho concentration (31). The most recent report showed the presence of oxidized LDL in human salivary samples and highlighted a correlation between salivary and serum oxidized LDL levels. Moreover, salivary oxidized LDL levels were higher in overweight/obese subjects than in control subjects (32).

In this study, we were able to quantify oxidized LDL levels in dogs and cats that had been diagnosed as healthy by their veterinarians. These levels were approximately one-quarter of the oxidized LDL value of healthy humans (11.2 ± 0.3 μg/dl) (29). Dogs and cats are known as high HDL animals, in that HDL is dominant, and the total amount of LDL is typically lower than that of humans. Naturally, oxidized LDL levels in dogs and cats are lower than that seen in humans. However, some obese dogs and cats showed extremely high oxidized LDL values. The oxidized LDL values in two obese dogs and three obese cats were indeed higher than the mean value in humans (20.1 ± 1.1 μg/dl) with coronary artery disease (29). Interestingly, the two high oxidized LDL dogs were in the BCS4 group, not the BCS5 group. The three high oxidized LDL cats were composed of one BCS4 cat and two BCS5 cats, and the highest oxidized LDL cat was in BCS4 group. This result suggests that BCS is not correlated with oxidized LDL levels.

Limitations of this study include a small sample size for the determination of reference ranges. Furthermore, the result of the LDL fraction migration by electrophoresis was not consistent with the ELISA results. In humans, migration of the LDL fraction peak toward the positive electrode is determined by the charge of the LDL particle, reflecting both glycation and oxidation. This is because the negative charge of apo B100 in LDL increases by modifications, such as oxidation and glycation (33). A previous human study reported that plasma could be separated into LDL sub-fractions [modified LDL (=oxidized LDL) and native slow-migrating LDL] by capillary electrophoresis, and oxidized LDL was shown as modified LDL. In this study, migration was not clearly visible via this electrophoresis method, and further studies using an appropriate electrophoresis method need to be performed.

Atherosclerosis is characterized by the accumulation of lipids and cholesterol in the inner membrane and tunica media of arteries (34). There are variations in lipoprotein metabolism among animal species, and dogs are less likely to develop atherosclerosis when compared to humans (35). Atherosclerosis is a cardiovascular and fibroproliferative inflammatory disease commonly associated with age- and dietary-related factors in humans (36). In a retrospective study of 21 dogs diagnosed with atherosclerosis on postmortem examinations, Miniature Schnauzer, Doberman pinscher, and Golden Retriever breeds were overrepresented, possibly owing to the increased risk for dyslipoproteinemia in these three breeds (37). Unfortunately, our obese high oxidized LDL dogs were Miniature dachshund and Mixed breed. In order to verify the breed predilection, more samples are needed.

Atherosclerosis in dogs occurs secondary to chronic inflammatory diseases, such as hypothyroidism (38), diabetes mellitus (39), hyperlipidemia (40), and dyspnea associated with obesity (34). These diseases usually affect the brain, eyes, spinal cord, heart, spleen, kidneys, lungs, pancreas, alimentary tract, and urogenital organs, such as prostate and urinary bladder (41). The association between atherosclerosis and glomerular disease is well established in humans (42). Interestingly, canine glomerulopathy is more prevalent in dogs with atherosclerosis as compared to dogs without atherosclerosis. Furthermore, canine atherosclerosis accompanied by glomerulopathy contributes to an elevation in serum cholesterol concentrations (27).

In this study, there was no significant difference in oxidized LDL values between normal and obese animals, whereas there were significant differences in T-Cho among BCS groups of dogs and cats. The correlation between T-Cho and oxidized LDL did not clearly show any trend. It suggests that obesity alone does not have a direct effect on serum oxidized LDL values in healthy dogs and cats. On measuring oxidized LDL by electrophoresis, we found that it is difficult to verify the migration of LDL fractions as modified LDL. In future studies, oxidized LDL will be analyzed by capillary electrophoresis, which will separate the LDL fractions more clearly.

The association between atherosclerosis and glomerular disease in humans is multifactorial. Human arteriosclerosis with renal failure is shown to progress rapidly and has not been fully understood. When renal failure is progressing, enhanced expression of LOX-1 (receptor for oxidized LDL) is involved in the formation of interstitial lesions in particular (43). However, oxidized LDL may be a risk factor for chronic diseases in animals, such as renal failure and diabetes mellitus. As a future study, we plan to investigate whether inflammatory diseases (i.e., diabetes mellitus, hypothyroidism, renal failure) can influence or induce different changes in oxidized LDL and/or cholesterol lipoprotein fraction ratios.

Unfortunately, this study has limitations. Only a small number of dogs and cats could be included in each group; hence, results and conclusions need to be interpreted with care. In order to validate our results, a larger sample pool is needed.

## Conflict of Interest Statement

The authors declare that the research was conducted in the absence of any commercial or financial relationships that could be construed as a potential conflict of interest.
